# A Novel Class of RIP1/RIP3 Dual Inhibitors

**Published:** 2020

**Authors:** Ting Zhou, Bo Liu

**Affiliations:** 1 Department of Surgery, School of Medicine and Public Health, University of Wisconsin-Madison, Madison, WI 53705, USA; 2 Department of Cellular and Regenerative Biology, School of Medicine and Public Health, University of Wisconsin-Madison, Madison, WI 53705, USA

Necroptosis is a form of programed necrosis mediated by receptor-interacting serine/threonine-protein kinase 1 (RIP1) and RIP3 and the subsequent phosphorylation of mixed lineage kinase domain-like protein (MLKL) [1–4]. Necroptosis has been implicated in multiple human diseases such as myocardial infarction, atherosclerosis, and abdominal aortic aneurysms (AAAs). Levels of RIP3 are elevated in the human tissues affected by these diseases. Furthermore, mice deficient of the gene encoding RIP3 are protected from disease in preclinical models [5–8].

Necroptosis and inflammation are intimately associated. Inflamed tissues often contain necrotic cells. Many pro-inflammatory cytokines such as TNFα are capable of triggering necroptosis [9]. Conversely, necroptosis is highly immunogenic due to the disruption of cellular membranes and the subsequent release of intracellular contents. Moreover, necroptosis mediators RIP1 and RIP3 can promote pathological inflammatory processes independent of their functions in cell death. For instance, inflammasome activation and IL-1 maturation in LPS-primed macrophages requires RIP3 [10]. In dendritic cells, caspase-8 deficiency facilitated LPS-induced inflammasome assembly which also depends on RIP3, as well as RIP1 [11]. Lawlor et al. confirmed the role of RIP3 in inflammasome and IL-1 maturation *in vivo* using a rheumatoid arthritis mouse model [12]. In our own study of vascular smooth muscle cells (SMCs), we demonstrated that RIP3 regulates the expression of pro-inflammatory cytokines and adhesion molecules through the NF-κB pathway [7]. Collectively, preclinical evidence accumulated during the past decades suggest that targeting RIP1 or/and RIP3 is likely to have therapeutic benefit in disease conditions involving cell necrosis and inflammation.

Since the discovery of RIP1 and RIP3 being key regulators in necroptosis and inflammation, multiple efforts have been devoted towards the development of small-molecule inhibitors that target these proteins. The first RIP1 inhibitor Necrostatin-1 (Nec-1) was discovered by Yuan’s group in 2005 [13]. Nec-1 and its modified version Nec-1s were shown to have protective effect in various murine disease models including neuronal loss, photoreceptor loss, ischemic brain injury, myocardial infarction, and atherosclerosis [5,13–16]. Using preclinical AAA models, our lab previously showed that administration of Nec-1 and Nec-1s attenuates aneurysm growth, and blocks progression of existing AAAs [17].

Compared to RIP1 inhibitors, the progress of developing pharmacological inhibition of RIP3 is slower. Mandal et al. identified a group of RIP3 inhibitors including GSK’872, GSK’840, and GSK’843 [18]. These compounds are highly selective to RIP3, but they induce apoptosis at high concentrations (>3 μM). Interestingly, the binding of GSK’843 or related compounds to RIP3 causes a kinase-independent conformation change of RIP3 that leads to the assembly of a pro-apoptotic complex [18]. This pro-apoptotic effect has limited the *in vivo* application of GSK’843 and similar compounds. However, Yang et al. reported that intraperitoneal (IP) administration of 1.9 mmol/kg GSK’872 alleviated ischemic stroke in murine middle cerebral artery occlusion model [19]. In the absence of pharmacological and toxicological data of the compound, it is unclear how well the mice tolerated GSK’872 in Yang’s study. However, in light of the pro-apoptotic effects reported by Mandal and colleagues, GSK’872 and similar compounds need to be systematically evaluated for potential toxicity in mice or other experimental models.

To seek a safer RIP3 inhibitor, we screened 1141 kinase inhibitors in mouse SMCs against necroptosis, and identified a group of compounds (represented by GSK’074) that met three selection criteria: 1) more potent than Nec-1s; 2) minimum cytotoxicity at 1 μM, and 3) low predicted docking energy to human RIP3. The IC50 of GSK’074 in the cell viability assay is 3 nM, which is in sharp contrast to 803 nM of Nec-1s. In addition to SMCs, GSK’074 is effective in multiple cell types including mouse fibroblast cell line L929 cells, mouse bone marrow derived macrophages, and human colorectal adenocarcinoma cell line HT-29 cells. The ability of GSK’074 to protect cells of different types and origins is important for drug development. A few necroptosis inhibitors such as GSK’481 (a RIP1 inhibitor) and necrosulfonamide (a MLKL inhibitor) are only effective in primate/human cells, preventing them to be tested in rodent models.

In this manuscript published in *Cell Death and Disease* [20], we presented biochemical and cellular data to demonstrate the molecular mechanism whereby GSK’074 attenuates necroptosis. At the cellular level, GSK’074 inhibits necroptosis by blocking interaction between RIP1 and RIP3, and the subsequent phosphorylation of MLKL. At the biochemical level, we conducted *in vitro* binding assay and kinase assay against RIP1 and RIP3. Interestingly, while GSK’074 binds to RIP3 with a disassociation constant (Kd) of 130 nM, it binds to RIP1 with higher affinity (Kd=12 nM). *In vitro* kinase assay confirmed GSK’074 inhibits kinase activities of both RIP1 and RIP3. Molecular docking further predicts that GSK’074 binds to RIP1 and RIP3 as type II kinase inhibitor in a DFG-out conformation (compound “locks” protein to an inactive state), which is different from other RIP3 inhibitors such as GSK’843 that is predicted binding to RIP3 as type I kinase inhibitor in a DFG-in conformation (compound competes with ATP at ATP binding site while protein is at an active state). Necroptosis commonly requires both RIP1 and RIP3, and therefore can be attenuated by inhibition of either kinase. However, interferon-β in the presence of poly(I:C) and zVAD activates toll-like receptor 3 and causes necroptosis that requires RIP3 but not RIP1 [18,20]. Similarly, RIP3 stimulates SMC inflammation independent of RIP1 [7,17]. Even in these RIP3-dependent RIP1-indpendent cellular processes, GSK’074 shows significant protective effects.

Since other RIP3 inhibitors (such as GSK’843 reported by Mandal et al.) induce apoptosis at high concentrations (>3 μM) [18], we carefully tested whether GSK’074 had pro-apoptotic effect. At a concentration range of 10 nM to 20 μM, GSK’074 does not induce apoptosis. We also explored the selectivity of GSK’074 among 403 human kinases. At 100 nM, four other kinases show similar binding affinity as RIP1 (KIT, MEK5, CSF1R, EPHB6). None of these four kinases have established functions in cell death; all are involved in oncogenic signaling. Whether GSK’074 could have an application in cancer therapy remains to be tested.

Encouraged by its minimum cytotoxicity and high potency against necroptosis and inflammation, we examined GSK’074 *in vivo*. When administrated orally, GSK’074 shows half-life of 2.4 hours in plasma. We used two distinct murine AAA models to determine the potential therapeutic effect of GSK’074. In the calcium phosphate induced AAA model, daily IP injection of 2 μmol/kg GSK’074 reduces necroptosis and apoptosis, diminishes macrophages infiltration, preserves SMCs viability, and attenuates aneurysm growth of young male C57BL/6J mice. In older ApoE deficient female mice, GSK’074 is also found to effectively block aneurysm formation triggered by Angiotensin II infusion.

In summary, our study identifies a novel class of necroptosis inhibitors represented by GSK’074 that inhibit both RIP1 and RIP3 as type II kinase inhibitors. Although we have only tested the therapeutic potential of the dual RIP1-RIP3 inhibitors in murine models of aortic aneurysm, GSK’074’s potent anti-cell death and anti-inflammatory effects make them a promising class of drug candidates. We believe that the discovery of these new inhibitors will pave the way for further pharmacological studies in AAAs and other diseases involving necroptosis and inflammation.
